# Concept about the Virulence Factor of *Legionella*

**DOI:** 10.3390/microorganisms11010074

**Published:** 2022-12-27

**Authors:** Jin-Lei Yang, Danyang Li, Xiao-Yong Zhan

**Affiliations:** The Seventh Affiliated Hospital, Sun Yat-sen University, Shenzhen 518107, China

**Keywords:** *Legionella*, virulence factor, infection, protozoans, humans, immune response

## Abstract

Pathogenic species of *Legionella* can infect human alveolar macrophages through *Legionella*-containing aerosols to cause a disease called Legionellosis, which has two forms: a flu-like Pontiac fever and severe pneumonia named Legionnaires’ disease (LD). *Legionella* is an opportunistic pathogen that frequently presents in aquatic environments as a biofilm or protozoa parasite. Long-term interaction and extensive co-evolution with various genera of amoebae render *Legionellae* pathogenic to infect humans and also generate virulence differentiation and heterogeneity. Conventionally, the proteins involved in initiating replication processes and human macrophage infections have been regarded as virulence factors and linked to pathogenicity. However, because some of the virulence factors are associated with the infection of protozoa and macrophages, it would be more accurate to classify them as survival factors rather than virulence factors. Given that the molecular basis of virulence variations among non-pathogenic, pathogenic, and highly pathogenic *Legionella* has not yet been elaborated from the perspective of virulence factors, a comprehensive explanation of how *Legionella* infects its natural hosts, protozoans, and accidental hosts, humans is essential to show a novel concept regarding the virulence factor of *Legionella.* In this review, we overviewed the pathogenic development of *Legionella* from protozoa, the function of conventional virulence factors in the infections of protozoa and macrophages, the host’s innate immune system, and factors involved in regulating the host immune response, before discussing a probably new definition for the virulence factors of *Legionella*.

## 1. Introduction

*Legionella* is a group of gram-negative bacteria with a facultative lifestyle, living as both intracellular parasites of free-living protozoa and in the form of biofilm with other microorganisms in the environment [1]. The bacteria have a ubiquitous distribution in natural environments. Aquatic environments, such as freshwater and moist soil are their major reservoirs, where their environmental hosts protozoa contribute to their ubiquitous distribution and dissemination in various aquatic environments by carrying them everywhere [2,3]. *Legionella* bacteria can enter a variety of man-made water systems, including domestic potable water systems and public infrastructure where they are more likely to contact humans [4]. Pathogenic *Legionella* species can infect human alveolar macrophages via *Legionella*-containing aerosols derived from the environment, resulting in Legionellosis, which manifests in two clinical forms: a self-limited disease (Pontiac fever) and a fatal severe pneumonia with various extrapulmonary features (Legionnaires’ disease, LD) [4,5]. LD shows a considerable mortality rate of about 8–10% overall. Despite the availability of effective antibacterial agents such as macrolides and quinolones, the elderly, smokers, people with chronic respiratory diseases, and immunocompromised people die at a higher rate [4,6]. The most common sources of outbreaks are bacteria-contaminated places such as potting soil, compost, public infrastructure, fountains, domestic water facilities, etc., and almost no person-to-person transmission cases are reported [7,8].

In 1977, the genus *Legionella* was first known when *Legionella pneumonia* (*L. pneumonia*) was recognized as the causative agent for a large outbreak of pneumonia that had occurred in Philadelphia in 1976 [9]. After that, many other *Legionella* species were consecutively identified, and now nearly half of the identified species (71 species, based on the LPSN—List of Prokaryotic Names with Standing in Nomenclature, www.bacterio.net (accessed on 22 December 2022)) are known to be responsible for nosocomial-acquired or community-acquired pneumonia [10,11], but not every pathogenic species makes the same contribution to LD cases. *L. pneumophila* ranks first, causing 91.5% of LD cases worldwide, with serogroup 1 (LP sg1) accounting for the largest proportion (84%) [12]. Other pathogenic species contribute less significantly to LD cases; for example, *Legionella bozemanae*, *Legionella micdadei*, and *Legionella longbeachae* are the second most common etiological agents, accounting for only about 2–7% of global Legionellosis cases [12,13]. A study of 140 sporadic cases of community-acquired pneumonia in Japan from December 2006 to March 2019 showed that the most frequently isolated species was *L. pneumophila* (90.7%), followed by *L. bozemanae* (3.6%), *L. dumofii* (3.6%), *L. micdadei* (1.4%), and *L. longbeachae* (0.7%) [14]. However, *L. longbeachae* was the most dominant species for legionellosis in New Zealand and Thailand in 2011, and legionellosis caused by *L. longbeachae* in Australia was second only to those caused by *L. pneumophila* (42% vs. 51%) from 1996 to 2000 [8,15]. The dominance of *L. pneumophila* and *L. longbeachae* as the prevalent pathogenic species infecting humans may be related to their environmental distribution, as *L. pneumophila* presents the most extensive distribution in multifarious water (84.50%) or soil (70.73%) sources [16], and *L. longbeachae* is frequently related to potting soil or compost [8,13,17]. The high proportion of LD cases caused by *L. pneumophila* and *L. longbeachae* indicates that they have stronger potential virulence than other species [18], which may also result from the virulence differences among species, while the molecular basis for discrepant pathogenicity of different species is unclear.

*Legionella* infects both its natural host, protozoa, and pathogenic host human macrophages via similar molecular mechanisms [19]. As a result, questions about how the potential pathogenicity of *Legionella* developed and what virulent heterogeneity exists between pathogenic and non-pathogenic *Legionella* arise. The pathogenicity of *Legionella* has long been considered to be related to its ability to invade human macrophages and replicate within cells. For bacterial infectivity, there is a preliminary insight into the virulence of *Legionella* through observing the transformation between virulent phases and non-virulent phases. In an environment with inadequate nourishment, *L. pneumophila* changes from a replicative phase (avirulent) to a transmissive phase (virulent), accompanied by increased motility and expression of multiple virulence factors, including flagella and the Dot/Icm type IV secretion system (Dot/Icm T4SS) [20]. In the transmissive stage, it tends to search for and invade a new host. This transformation can also be seen in the expression of macrophage infectivity potentiator (Mip), a peptidylproline *cis-trans*-isomerase on the membrane surface that is needed for adherence and invasion of new host cells as a marker of virulence, whose expression is remarkably repressed in the early stage of infection and predominantly up-regulated at the late stage of infection [21]. Similar oscillation in *mip* gene expression also occurs in *L. pneumophila* from early to late stage biofilm formation [22]. Highly pathogenic strains are provided with not only potent invasive ability but also robust replication capacity within human macrophages, as evidenced by the finding that some *L. pneumophila* mutants that are avirulent for humans can survive but cannot multiply in phagolysosomes [23]. Mammalian cells are useful models for studying the intracellular multiplication of *Legionella* in eukaryotic cells, where the knowledge gained can be easily used to understand their pathogenic mechanism. For instance, *L. pneumophila* exhibits efficient invasion and consistent multiplication over 48 h after infecting THP-1 in vitro, while *L. feeleii* and *L. anisa* cause a mild flu-like illness named Pontiac fever and shows multiplication attenuation and diminished survival time after infecting THP-1 in vitro, and these species are avirulent in guinea pigs [24,25]. However, LfPF (*L. feeleii* ATCC 35072) causes Pontiac fever and exhibits milder virulence in a distinct manner, as it could multiply in human host cells [26], indicating that there are factors contributing to bacterial virulence other than intrusion and replication capabilities.

The molecular mechanism of *Legionella* pathogenicity focuses on those molecules required for invasion and replication. Gene mutant studies using protozoans and mammalian cells as cellular models identified many virulence-related proteins involved in the whole infection cycle. For example, a variety of *L. pneumophila* surface structures have been identified to be implicated in pathogenesis, including lipopolysaccharides (LPS), flagella, pili, outer membrane porin, Mip, outer membrane protein, and a 60-kDa heat shock protein (Hsp60). They all participate in the invasion of host cells [27]. Secreted factors from essential secretion systems are involved in altering the host cell signal pathways and regulating the host-pathogen interactions required for optimal replication. These factors include degradative enzymes that are secreted via the type II protein secretion system (T2SS), such as proteases, RNase, phosphatases, phospholipases, etc. and more than 300 effector proteins delivered by the Dot/Icm T4SS [28]. All of these proteins have been known as virulence factors. However, notably, *L. pneumophila* mutants with those genes essential for infectivity or replication within human macrophages also severely attenuated bacterial infectivity and intracellular survival in protozoans [29,30]. That is, rather than being specifically pathogenetic, these so-called virulence factors function in both natural and accidental host infections and are required for bacterial survival.

Recently, several articles showed that the virulence of *L. pneumophila* strains with invasion and proliferation ability is positively correlated with the level of NF-κB activation in the host after infection, and those strains that can induce a higher NF-κB activation level in vitro lead to more weight losses, higher mortality, more serious pathological phenotypes, and higher levels of serum cytokine production in A/J mice [31,32]. These findings suggest that *Legionella* virulence is characterized by a specific inflammatory response in the host triggered by pathogen-host interaction, in addition to the ability to infect human macrophages and carry out intracellular replication. Therefore, an update on the concept of the virulence factor of *Legionella* is required and needs to be detailed and documented.

## 2. *Legionella* Pathogenicity Evolution during Long-Term Co-Evolution with Protozoa

Although *Legionella* is well known as an intracellular pathogen of macrophages, it is originally a parasite of environmental protozoa. The intracellular replication within protozoa was first described in *L. pneumophila* in 1980 [33]. Studies on the interaction of *L. pneumophila* with environmental amoebae demonstrated that *Acanthamoeba* spp. [34,35,36], *Naegleria* spp. [37], *Hartmannella* spp. and *Balamuthia mandrillaris* support the intracellular growth of *L. pneumophila* [38]. Until now, at least 20 species of amoebae, two species of ciliated protozoa, and one species of *Slime mould* have been identified as potential environmental hosts for *Legionella* spp. [39]. As a group of facultatively intracellular parasites, *Legionella* has developed a dependent relationship with various protozoa that shelters them from stresses in harsh or low-nutrient environments. Indeed, when nutrients are scarce, free-living protozoa can support *Legionella* multiplication and help resuscitate viable non-culturable *L. pneumophila* after disinfection [40,41]. Protozoa are environmental hosts of *Legionella* species; they share many commonalities with mammalian phagocytes in microbicidal mechanisms, such as uptake and intracellular trafficking of *Legionella*, and they have been proposed as training grounds where parasitic microorganisms can exchange genes across species or with the amoeba hosts [42]. It was speculated that a long-term evolutionary connection with an amoeba host facilitates *Legionella’s* development of virulence strategies applied to escape routine digestive pathways in mammalian phagocytes [43]. Humans may be an evolutionary dead end and play little role in *Legionella* transmission, as the majority of cases of the disease had an environmental source.

*Legionella* can be easily consumed in the environment by free-living amoebae that feed on the biofilm containing *Legionella* and other multiple microorganisms [44]. Protozoa uptake bacteria through traditional or coiling phagocytosis and confine them to phagosomes that subsequently fuse with lysosomes. Undoubtedly, *Legionella* has evolved many clever strategies to escape from host digestion and then furtively replicate within the protozoan predator. The strategies include disrupting endocytic transport, preventing vacuolar acidification, and intervening in host metabolic pathways, such as protein translation and ubiquitination, phosphoinositide lipid metabolism, and so on [45,46,47]. Transmission electron microscopy showed that the *L. pneumophila*-containing phagosomes did not enter the routine digestive pathway and fuse with lysosomes. On the contrary, they recruit components of the rough endoplasmic reticulum and mitochondrion to build *Legionella*-containing vesicles (LCV) to replicate within host cells [48]. The infection process is mainly composed of five parts: bacterial uptake, the establishment of the LCV, intracellular multiplication, host response, and bacterial release [48]. The same procedure also takes place when it invades human cells, and the same several secretion systems are involved [49]. It is considered that the ability of *Legionella* to invade human macrophages is a consequence of its long-term adaptation to intracellular survival and multiplication in protozoa, as in the process of infection and survival within macrophages, *Legionella* shares some common mechanisms and genes with environmental hosts, such as effectors of Icm/Dot secretion system [50]. Therefore, human macrophages are assumed to be an unexpectedly accidental host in the long common evolutionary history of *Legionella*.

Many analyses of the *Legionella* genome have given insight into the signs of co-evolution between *Legionella* and protozoa. Firstly, *Legionella* spp. genomes have markedly high plasticity and diversity. Comparative genomics analysis of several *L. pneumophila* strains revealed that ~3000 proteins are coded and nearly 300 genes (10%) are specific for each strain [51]. This is unexpected for the same bacterial species. There are higher genomic divergences among different *Legionella* species. For example, *L. longbeachae* has 34.8% more specific genes compared with *L. pneumophila* strain *Paris*, *Lens*, *Philadelphia*, and *Corby*, and only 65.2% of genes are orthologous to them [52]. Genome sequence analysis based on 58 species showed that the genome size and GC content of *Legionella* are highly diverse, with varied ranges from 2.37 Mb to 4.88 Mb (*L. adelaidensis* to *L. santicrucis*) and 34.82% to 50.93% (*L. busanensis* to *L. geestiana*), respectively. Up to 32% of 5832 orthologous genes are strain-specific, and genes in the core genome account for only 6%, which is similar to the result obtained by analyzing 38 *Legionella* species [53,54]. The highly dynamic nature of these genomes implies that the *Legionella* genome can obtain genetic materials via other means than just vertical inheritance. Secondly, a high number of conserved eukaryotic-like proteins and virulence genes that functionally mimic host cell proteins were identified in the genome of *L. pneumophila* but were more conserved or absent in non-*L. pneumophila* [51,55,56]. Some of these proteins, which were found in more than two-thirds of the 58 analyzed species, contain F-box and U-box domains that have roles in interfering with eukaryotic ubiquitination machinery and regulating the proteasomal degradation pathway [53,57]. Another interesting finding is that some proteins have ankyrin-repeat, which targets the plasma membrane or the ER of eukaryotic cells, and is essential for the intracellular proliferation of *Legionella* in macrophages and protozoa [58,59]. Some eukaryote-homologous enzymes involved in metabolism and signaling pathways, such as Rho-, Ras-, or Rab-like proteins and phospholipases, are present in the genome of *Legionella* as well [53]. They are highly conserved among *L. pneumophila* genomes [60]. In addition, a vast majority of effectors translocated by the Dot/Icm type IV secretion system (Dot/Icm T4SS) are eukaryotic-homologous or have eukaryotic-like domains. All of them play important roles in the intracellular replication of human macrophages and protozoa [61]. Although eukaryotic-like proteins have been identified in many other bacterial pathogens, *L. pneumophila* has been shown to encode the largest and most extensive variety of eukaryotic-like proteins or proteins with eukaryotic-homologous domains [53]. A large number of eukaryotic-like proteins provide the bacteria with high versatility and the ability to adapt to intracellular conditions of eukaryotic cells, allowing them to infect a variety of hosts such as amoebas and humans.

The inconsistent GC content between eukaryotic-homologous proteins and genomes implies that horizontal gene transfer (HGT) from protozoa to *Legionella* may exist [43]. Moliner et al. have given an instance of gene interchange between *Dictyostelium discoideum* and *L. drancourtii* through a phylogenetic analysis of the malatesynthase, which shows the two are clustered with a bootstrap value of 88% [62]. Structural studies confirmed that a series of effector proteins secreted by Dot/Icm T4SS are acquired through interdomain HGT. An obvious example is RalF, an ADP-ribosylation factor guanine nucleotide exchange factor (GEF) transported through the type IV secretion apparatus, which comprises a Sec7 domain homologous to mammalian [63], and helps recruit Arf to *Legionella*-containing phagosomes for the establishment of a replicative organelle, LCV. Felipe et al. screened 62 eukaryotic-like genes of *Legionella* and found a considerable number of Dot/Icm T4SS substrates, with some eukaryotic domains such as ankyrin repeats, Leucine-rich repeats, F-/U-box, Ser/Thr kinase, coiled coils, etc. [56]. Another interesting example demonstrating the existence of HGT is the presence of sphingolipid metabolic enzymes, including sphingomyelinase, sphingosine kinase, and sphingosine-1 phosphate lyase in *L. pneumophila* genome. Sphingolipids are major components of eukaryotic cellular membranes and are generally conserved among all eukaryotes, but these enzymes appear to be evolutionarily conserved among several *Legionella* species [64,65]. All these examples illustrate that HGT occurs from protozoans to *Legionella*, and protozoans are the most predominant donors of eukaryotic-like proteins for *Legionella*, but they are not the only ones. Gene transfer via HGT occurs not only from protozoa to *Legionella*, but also among different *Legionella* species. A regulator-effectors island encoding a LuxR type regulator RegK3 and two Do/Icm T4SS effectors LegK3 and CegK3 was proved the presence of HGT among the genus *Legionella*, since the regK3-legK3-cegK3 island presented in different species as a whole and no single *Legionella* or *L. pneumophila* strain was found to harbor only one or two of these genes, but not all strains in the same species contain this genomic island. The regK3-legK3-cegK3 island shows a different phylogenetic tree topology within the *Legionella* species and has a lower GC content compared with the genomic GC content of the *Legionella* species [66]. The F-type T4ASS encodes a complete T4SS core as well as the necessary protein for pilus assembly and mating pair stabilization, shows homology and collinearity with the tra-region in *Escherichia coli* F plasmid and *Rickettsia belii*. The tra-region in the *L. pneumophila* strain *Paris* plasmid (Tra1) shows the most identity with that located on the *L. longbeachae* plasmid (Tra4) when compared with other *L. pneumophila* strains [60]. In addition, *L. pneumophila* also encodes proteins homologous to viruses that can infect amoeba and amoeba-associated bacteria [67]. HGT is a crucial way of providing eukaryotic-like proteins to *Legionella*. It occurs not only between the same or closely related species but also between different domains of the organism. Phylogenetic analysis on the evolutionary origin of eukaryotic-like proteins in *L. pneumophila* suggested that a portion of these proteins were acquired from eukaryotes, while several proteins containing a typically eukaryotic domain pertained to bacterial phylogeny [68]. An evolutionary dissection based on comparative genomics for Dot/Icm T4SS proteins suggested that both recombination and natural positive/negative selection are evolutionary forces that shape the diversity of Dot/Icm T4SS effectors [69,70]. As a result, lateral gene transfer and gradually convergent mutation that adapts to intracellular conditions both contribute to the pooling of eukaryotic-like proteins.

## 3. A Supplement to the Concept of Virulence Factors in *Legionella*

Virulence factors have always been defined as effectors and proteins contributing to any process of infection of human macrophages, whether in the bacterial invasion, hijacking vesicle transport, evasion autophagy, or replication. This definition originated from the perspective of humans and intends to understand the pathogenic mechanisms of *Legionella* [71]. However, it appears that a common concept for environmental and accidental hosts is required, as these factors are not only involved in the infection of human macrophages but also in protozoa natural parasitization (Table 1). Some contradictory things happened when those non-pathogenic *Legionella* species in humans also parasitizes protozoa in the environments, and effectors involved in or required in the infection of protozoa may partially overlap with those in pathogenic species [3]. *Legionella* has obtained a large number of redundant proteins that perform the same functions [72], which means some effectors execute parallel functions in different hosts, or there are many candidates to perform the same functions in one host [73]. Consequently, a complicated problem arises: how to clearly distinguish the range of virulence factors? More importantly, it is noteworthy that the pathogenicity of virulent *L. pneumophila* may be determined by elements other than the so-called virulence-related factors, such as viability or vitality, stability, and stress resistance [74]. In other words, a *Legionella* strain may be non-virulent or weakly virulent to humans even if it expresses all the virulence factors that are required to infect humans. Here, we take several traditional virulence factors, including components and effectors from secretion apparatuses as well as surface proteins as examples to discuss the role of these virulence factors in natural hosts and humans, and hopefully to propose a more specific definition of virulence factors.

The pathogenicity of *Legionella* is manifested as host cell invasion and building LCV for robust intracellular replication. The pathogenesis involves attachment to host cells and modulation of host metabolism, vesicle trafficking, host autophagy, protein translation, and degradation. Proteins required in these procedures are recognized as virulence factors, mainly including some structures and proteins on the surface of bacteria and effectors translocated by five secretion systems (the Dot/Icm T4SS, Lsp type II (T2SS), Tat, Lss type I (T1SS), and Lvh type IVA secretion systems (T4ASS) [28]. The five secretion systems’ apparatuses play important roles in the life cycle of *Legionella*. The Dot/Icm T4SS apparatus which localizes in the polar regions of bacterial cells and is composed of about 27 components, is recognized as a key virulence factor required for intracellular bacterial survival. It participates in almost the whole infection process after bacterial entry, including subverting vesicle trafficking, establishing the LCV, multiplication, inhibiting host cell apoptosis, and promoting bacterial release from host cells. The Dot/Icm T4SS is proposed to be particularly crucial for virulence-related phenotypes in the infection of human macrophages and amoebae because nearly 300 effector proteins that are essential for the regulation of host physiological and biochemical activities are translocated by it [28]. As a supplement to the Dot/Icm T4SS, the Lvh T4ASS conditionally works in bacterial entry and intracellular multiplication while delaying phagosome acidification [75]. The Lsp T2SS transports proteins from the periplasm to the extracellular space. Unfolded proteins are first translocated across the bacterial inner membrane by the general secretory (Sec) pathway, then secreted to extracellular space by T2SS after folding into a tertiary conformation. Some proteins that are folded within the cytoplasm and transported across the bacterial inner membrane via the twin-arginine translocation (Tat) system can also be secreted by the T2SS apparatus [76]. The Tat system contributes to biofilm formation and intracellular multiplication of *Legionella*. The Lss sT1SS is encoded by the lssXYZABD locus, among which the *lssB* and *lssD* genes encode the ABC transporter and membrane fusion protein, respectively. Although the relationship between T1SS and host-pathogen interactions has not been elaborated, *lssB* and *lssD* genes were demonstrated to be required for intracellular replication, as Δ*lssBD* mutant led to reduced internalization, replication delay and comprised cytotoxicity in amoebas [77]. The secreation systems’ apparatuses are defined as “virulence factors” due to their basic roles in infecting various hosts, however, they participate in both infection of natural and accidental hosts and play essential roles in the life cycle of *Legionella*. The secretion apparatus’s core components are essential for survival and necessities in the *Legionella* lifecycle.

Besides the core secretion apparatuses, effectors secreted by these systems are also required for the infection of natural hosts and accidental hosts. Among them, Dot/Icm T4SS and Lsp T2SS are the primary and essential apparatuses for invasion and intracellular replication. Most effectors secreted by the two secretion systems have been explored in terms of function and pathogenic implications and have been summarized in many articles [27,28,45,78]. Although some effectors play important roles in the infection of human macrophages, they may be indispensable for survival relying on the infection of natural hosts. Some *Legionella* with effector gene mutants presents intracellular growth defects in both human cells and protozoa. A new substrate, RavY translocated by Dot/Icm T4SS, was characterized as an important effector for maintaining intracellular replication of *L. pneumophila* in mammalian cells [79], and also functions in *L. pneumophila* replication within protozoan hosts [80]. The protein SdhA maintains the integrity of LCV to prevent cytoplasmic degradation and host cell death by blocking the action of inositol 5-phosphatase OCRL and controlling endosomal dynamics [81,82], which is necessary for virulence because a damaged LCV membrane will expose the bacteria to the cytoplasm, and activate cell pyroptosis as well as premature termination of the bacterial replication process. However, it can be easily speculated that SdhA undertakes the same functions essential for living in protozoa, given that *Legionella* escapes the endocytosis pathways of protozoa and macrophages, though a phenotype defect of Δ*sdhA* mutant in protozoa has not been reported. LCVs with the MavE effector mutant are unable to hijack ER-derived vesicles and fuse with lysosomes, resulting in defective growth in human monocyte-derived macrophages and amoebae and aborted intrapulmonary proliferation in mice [83]. SidJ is also a substrate of the Dot/Icm T4SS and is necessary for recruiting endoplasmic reticulum (ER) proteins and incorporating ER-generated vesicles, which are constantly expressed during the entire life cycle of *Legionella* [84]. Moreover, LegC2, LegC3, and LegC7 effectors with functions similar to SidJ have been described as “SNARE-like” proteins engaging in ER-derived membrane recruitment and fusion, but not participating in bacterial replication [85]. The DrrA protein targets Rab1 on the plasma membrane-derived vacuoles by displacing Rab-GDI to recruit vacuoles containing *Legionella* [86]. Undoubtedly, these proteins are essential to LCV building in both protozoa and macrophages. In addition, the gene *pmiA* outside the icm/dot loci was defined as a virulence factor of *L. pneumophila*, involved in the survival and replication of *L. pneumophila* in human macrophages and protozoa through avoiding the acquisition of the late endosomal-lysosomal markers LAMP-1 and LAMP-2, and mainly contributing to the latter [30], thus, it is more suitable for these elements to be called survival factors than virulence factors. The effectors stated above involve both protozoa and human infection; nevertheless, infecting natural hosts is their original and primary feature; their pathogenicity to humans is an unexpected result of their evolution in order to survive in diverse environmental hosts and accidentally adapt to the intracellular environment of macrophages. And infecting humans is not the primary way of life for *Legionella* but the point of death. Therefore, to some extent, they should not be defined as virulence factors that are specific to humans.

Many surface proteins involved in invading host cells are also identified as virulence factors through genomic DNA library screening, gene complementation experiments, and defective phenotypes of mutants observed after the infection of macrophages or amoebae [87]. Protozoa and macrophages are valuable cellular models commonly used in exploring the functions of *Legionella* proteins. For instance, *rtxA* and *enhC* loci were screened by transposon mutagenesis with significantly reduced entry into host cells compared with wild-type strains [87]. RtxA is homologous to repeats structural toxin protein secreted by T1SS, is associated with cytotoxic activity on the bacterial surface, and is required for bacterial entry via binding β2 integrins [88,89]. EnhC is a secreted protein with Sel1 repeat (SLR) motifs that can interact with eukaryotic proteins possessing immunoglobulin-like folds. The *rtxA* gene was demonstrated to be virulence-related by an in-frame deletion in the gene, and its product is a modular multifunctional protein involved in multiple infection steps, including adherence of the host, cytotoxicity, pore formation, intracellular growth, damage to mice, and especially cell entry [90,91]. Similar functions of *rtxA* could also be observed when using the environmental host *Acanthamoeba castellanii* as a cellular model [92], suggesting that *rtxA* plays a role in cell entry and replication in both macrophages and protozoa. In contrast, the *enhC* locus was demonstrated to be dispensable for uptake into host cells and required for robust replication within macrophages but not *Dictyostelium discoideum*, which may result from the EnhC protein releasing innate immune pressure from infected macrophages, since soluble factors secreted by infected monolayers restrict intracellular growth of Δ*enhC L. pneumophila*, and the growth restriction caused by low concentrations of recombinant mouse TNF-α could be compensated by a plasmid-encoded EnhC [93]. Similarly, LpnE is another protein with SLR regions, it can moderately enhance infection in both protozoa and mouse models and regulate vesicle trafficking for the avoidance of LAMP-1. But this protein seems to play a more important role in the infection of macrophages because LpnE mutant *L. pneumophila* showed significantly attenuated multiplication in the lungs of A/J mice after a period of infection, which may be caused by compromised intrusion and avoidance of lysosomal digestion, or more likely by the weakened resistance to innate immunity in mice like those infected with Δ*enhC L. pneumophila*, because comparable bacterial numbers were obtained by either the LpnE mutant or the wild type strain 72 h after amoeba infection [94]. EnhC participates in immune escape and the persistent survival of *Legionella* against human macrophages but not protozoa, while LpnE plays a pivotal role in the continuous growth and proliferation of *L. pneumophila* in A/J mice but not in amoeba. Therefore, they are more specific virulence factors for susceptible mammals, such as humans, but not necessary factors for infecting protozoa. Other bacterial surface proteins, such as flagella and type IV pilin, play equally important roles in adherence to human cells and protozoa, as well as foreign antigens involved in routine immunity [95]. As an adhesion molecule, the major outer membrane protein (MOMP) helps *L. pneumophila* bind to macrophages and initiate an immune response [96]. Although the above three proteins (EnhC, LpnE, and MOMP) assist bacterial entry, they act as conventional antigens causing a host immune response; they are not virulence factors specific to humans. The most typically pathogenicity-related protein on the bacterial surface is the macrophage infectivity potentiator (Mip), a Peptidyl-prolyl-cis/trans-isomerase (PPIase), which can bind to collagen IV and change peptidyl-prolyl bonds to help bacteria transmigrate through the barrier of extracellular matrix (ECM) and NCI-H292 lung epithelial cells [97]. Although the Mip protein increases bacterial infectivity to protozoa and macrophages [98], the molecular structures involved are not the same. The N-terminal and dimerization of Mip are necessary for optimal virulence in amoebas, while PPIase activity is significantly important for the infection of animal models [99]. Despite having a role in adaptation to intracellular growth within protozoa, Mip has evolved a specific molecular mechanism for infecting animals or humans.

**Table 1:** *Legionella* molecules participate in the infection of protozoans and humans.

Classification	Molecule	Coding Gene	Function in Infection of Protozoa	Function in Infection of Humans	Regulate Host Immune Response	Virulence Factor Specific to Protozoa	Ref.
**Bacterial surface structure**							
1	EnhC	*enhC*	Absence has no influence in *Legionella* growth within the amoeba	Adhesive molecule for host cells; trafficking of the *Legionella*-containing vacuole; decreasing NF-κB activation	Yes	Yes	[93,96,100]
2	Lcl	*lcl*	Inducing *Legionella* autoaggregation promotes the infection of protozoa	A polymorphic adhesin of *L. pneumophila* that binds to immunogenic GAG and human lung epithelial cells	No, it is antigenic in human infection.	No	[101,102,103]
3	Hsp60	*htpB*	N/A	Promoting attachment and invasion.	No	No	[104,105]
4	MOMP	*mompS*	N/A	Mediating phagocytosis via the macrophage integrin receptors CR1 and CR3	No	No	[106]
5	type IV pili	*pilB-E*	Adherence of host cells	Adherence of host cells	No	No	[95,107]
6	Rtx	*rtx*	Adherence; intracellular survival and trafficking	Adherence of host cells; helping entry to host cells; affecting cytotoxicity and pore formation, is required for optimal replication of *Legionella*	No	No	[87,90]
7	LadC	*ladC*	Adherence; assisting bacterial replication	Adherence of host cells; assisting bacterial replication	No	No	[108]
8	flagellin	*flaA*	Invasion; multiplication	Facilitating the encounter of the host cell; enhancing the invasion capacity; and inducing caspase-1-mediated macrophage death	No	No	[109,110,111]
9	Mip	*mip*	No effect on adherence and initial interactions; involved in bacterial resistance to intracellular killing and/or intracellular multiplication.	Bacterial penetration of the lung epithelial barrier; infection of the host cell; contributing to the intracellular survival of *Legionellae*; necessary for full virulence	No	Yes, having eukaryotic homologous structure for breaking lung epithelial barrier.	[112,113,114]
**T4SS effectors**							
1	LpdA	*lpdA*	A phospholipase-D; intracellular multiplication	Modulating the lipid composition of the LCV, affecting the distribution of diacylglycerol and phosphatidic acid; disruptiing the Golgi apparatus; optimal survival of *Legionella*	No	No	[115,116]
2	LecE	*lecE*	A phospholipase-D; intracellular multiplication	Affecting distribution of diacylglycerol and phosphatidic acid	No	No	[115]
3	LegC7, LegC2 and LegC3	*ylfA*, *ylfB* and *legC3*	N/A	Assembling a complex on the LCV to initiate membrane fusion with ER-derived vesicles	No	No	[83]
4	MavC	*mavC*	N/A	Inhibiting host immunity by mediating the ubiquitination of E2 enzyme UBE2N in the initial phase of bacterial infection	Yes, directly	Yes	[117]
5	MavE	*mavE*	Essential for intracellular growth of *Legionella*; fusing LCV with endoplasmic reticulum (ER)-derived vesicles.	Essential for intracellular growth in human monocyte-derived macrophages and intrapulmonary proliferation in mice; fusing LCV with ER-derived vesicles; phagosome biogenesis and lysosomal evasion	No	No	[83]
6	Lgt family	* lgt1-3 *	A cytotoxic glucosyltransferase.	Glycosylates the mammalian elongation factor eEF1A and inhibits its activity; affecting the protein synthesis of host cells.	Yes, indirectly	No	[118]
7	LegK1	*legK1*	N/A	An activator of NF-κB	Yes, directly	Yes	[119]
8	LegK2-4	*legK2-4*	Influence the intracellular replication and growth rate of bacteria; LegK2 plays role in the cytotoxicity of *Legionella* toward amoeba	Kinases inhibit actin nucleation on phagosomes to interrupt late endosome/lysosome trafficking to the LCV	No	No	[120]
9	LegG1	*legG1*	A Ran GTPase activator; inhibition migration and chemotaxis of protozoa	A Ran GTPase activator localizing to the LCV membrane; enhancing LCV motility by stabilization or increased polymerization of microtubules and promoting intracellular bacterial replication in phagocytes; modulating phagocyte migration	Yes, indirectly.	No	[121,122]
10	LegAS4	*legAS4*	Promoting rDNA transcription	Promoting rDNA transcription	Yes, indirectly	No	[123]
11	LnaB	*lnaB*	N/A	An activator of NF-κB	Yes, directly	Yes	[119]
12	SidC	*sidC*	N/A	subversion of host vesicular transport; participation in the recruitment of Arf1 and ubiquitin to the LCV; utilization of the ubiquitin and phosphoinositide pathways; functions in LCV establishment and is required for optimal growth	No	No	[47,124]
13	SdcA	*sdcA*	N/A	Functions in LCV establishment and is required for optimal growth; recruiting Arf1 and ubiquitin to the LCV	No	No	[124]
14	SidD	*sidD*	A deAMPylase modifying Rab1; reversing the effect of SidM	A deAMPylase modifying Rab1; reversing the effect of SidM	No	No	[125]
15	SdeA, SdeB, SdeC and SidE	*sdeA*, *sdeB*, *sdeC* and *sidE*	SidE family, phosphoribosyl ubiquitination ligases ubiquitinate multiple ER membrane and Golgi resident proteins such as Rab small GTPases to rearrange tubular ER and induce Golgi fragmentation	SidE family members. Phosphoribosyl ubiquitination ligases; ubiquitinate multiple ER membranes and Golgi resident proteins such as Rab small GTPases to rearrange tubular ER and induce Golgi fragmentation.	No	No	[126,127]
16	SidK	*sidK*	Inhibiting host vacuolar H+-ATPase to maintain a neutral pH in the phagosome	Inhibiting host vacuolar H+-ATPase to maintain a neutral pH in the phagosome	No	No	[128]
17	SidJ	*sidJ*	CaM-activated glutamylase for SidE	CaM-activated glutamylase for SidE	No	No	[129]
18	SidP	*sidP*	Phosphoinositide-3-phosphatase; subverting host cell phosphoinositide (PI) metabolism	Phosphoinositide-3-phosphatase; subverting host cell phosphoinositide (PI) metabolism	No	No	[130]
19	SidM/ DrrA	*drrA*	Recruiting and activating small GTPase Rab1; no influence in intracellular growth of *Legionella*	Recruiting small GTPase Rab1 and locking its activation of by guanine nucleotide exchange factor (GEF) and AMPylation activity; no influence in intracellular growth of *Legionella*	No	No	[131]
20	AnkB	*ankB*	Polyubiquitination of proteins; decoration of the LCV with polyubiquitinated proteins; promoting intracellular proliferation	Polyubiquitination of proteins; decoration of the LCV with polyubiquitinated proteins; promoting intracellular proliferation	No	No	[59]
21	VirD4	*virD4*	A coupling protein for the Lvh T4ASS	A coupling protein for the Lvh T4ASS	No	No	[132]
22	LpnE	*lpnE*	Required for efficient infection of amoeba	Helping bacterial entry; influencing host trafficking of *Legionella* vacuole	No	No	[94,133]
23	NudA	*nudA*	A Nudix hydrolase; promoting growth	A Nudix hydrolase; promoting growth	No	No	[134]
24	PsrA	*psrA*	Helping bacterial intracellular growth in protozoa	Dispensable for growth in human macrophages.	No	No	[135]
25	Lem10	*Lem10*	Non-catalytic HD domains-containing protein	Non-catalytic HD domain-containing protein	N/A	N/A	[136]
26	RavY	*ravY*	N/A	Essential for promoting intracellular replication but not survival.	No	No	[79]
27	MavE	*mavE*	Intracellular growth	Intracellular growth; remodeling the LCV with ER-derived vesicles; preventing LCV from fusing with lysosome	No	No	[83]
28	MavL	*mavL*	N/A	As a signaling protein that binds ADP-ribose; interacting with mammalian ubiquitin-conjugating enzyme UBE2Q1	N/A	N/A	[137]
29	MavQ	*mavQ*	A phosphatidylinositol (PI) 3-kinase targeting to ER; driving rapid PI3-phosphate turnover on the ER and remodeling of host ER membrane	A phosphatidylinositol (PI) 3-kinase targeting to ER; driving rapid PI3-phosphate turnover on the ER and remodeling of host ER membrane	No	No	[138]
30	LtpD	*ltpD*	N/A	Bound directly to phosphatidylinositol 3-phosphate and inositol (myo)-1 (or 4)-monophosphatase 1; involved in bacterial replication; modulating endocytic vesicle traffic	N/A	N/A	[139]
31	LtpM	*ltpM*	A glycosyltransferase; dispensable for replication	A glycosyltransferase; dispensable for replication	N/A	N/A	[140]
32	SetA	*setA*	O-glucosyltransferase	O-glucosyltransferase; causes a robust nuclear translocation of transcription factor EB	N/A	N/A	[141,142]
33	RidL	*ridL*	Hijacking the host scaffold protein VPS29 critical for endosomal cargo recycling; inhibiting retrograde trafficking; promoting intracellular bacterial replication	Hijacking the host scaffold protein VPS29 critical for endosomal cargo recycling; inhibiting retrograde trafficking; promoting intracellular bacterial replication	No	No	[143]
34	SidI	*sidI*	N/A	Interacting with the host translation factor eEF1A and eEF1Bgamma; inhibiting eukaryotic protein translation; inducing host stress response	Yes, indirectly	No	[144]
35	MesI	*mesI*	N/A	Inhibiting the activity of SidI; required for optimal intracellular bacterial replication.	Yes, indirectly.	No	[145]
36	Sde family	*sdes*	Phosphoribosyl-linked ubiquitination	Phosphoribosyl-linked ubiquitination; resulting in tubule rearrangements of ER	N/A	N/A	[126,146]
37	PieE	*pieE*	N/A	PieE localized to the endoplasmic reticulum (ER) and induced the formation of organized smooth ER	N/A	N/A	[147]
38	SdhA	*sdhA*	Maintaining LCV integrity	Maintaining LCV integrity	Yes, indirectly.	No	[148]
39	Lart1	*lart1*	Directly targets NAD+-dependent glutamate dehydrogenase (GDH) enzymes	N/A	N/A	N/A	[149]
40	RavD	*ravD*	No effects on intracellular growth; suppressing endolysosomal maturation	No effects on intracellular growth; suppressing endolysosomal maturation; inhibiting the NF-κB pathway via deubiquitinase activity exquisitely specific for linear Ub chains	Yes, directly.	Yes	[150,151]
41	LegG1	*legG1*	A Ran GTPase activator; inhibiting *D. discoideum* migration and stimulating cell motility	A Ran GTPase activator; inhibiting phagocyte migration and chemotaxis	Yes, indirectly.	No	[122]
42	RavK	*ravK*	N/A	Cleaving actin and disrupting host cytoskeletal structure	N/A	N/A	[152]
43	PlcA-C	*plcA-C*	Zn (2+)-dependent PLC family; essential for intracellular replication	Zn (2+)-dependent PLC family; essential for intracellular replication	N/A	N/A	[153]
44	Ceg3	*ceg3*	A mono-ADP-ribosyltransferase that localizes to the host mitochondria, regulating host energy metabolism; not affect the intracellular replication.	A mono-ADP-ribosyltransferase that localizes to the host mitochondria, regulating host energy metabolism; not affect the intracellular replication	N/A	N/A	[154]
45	Ceg4	*ceg4*	N/A	A phosphotyrosine phosphatase decreasing activation of eukaryotic MAPK pathways	Yes, directly.	Yes	[155]
46	VipA	*vipA*	An actin nucleator that enhancesactin polymerization; altering host cell organelle trafficking; not essential for bacterial entry or replication	An actin nucleator that enhances actin polymerization; altering host cell organelle trafficking; not essential for bacterial entry or replication	No	No	[156]
47	SdbA	*sdbA*	N/A	Be important for bacterial replication; continuous activation of NF-κB	Yes, directly.	Yes	[157]
48	SdcA	*sdcA*	Bacterial E3 ubiquitin ligases as a paralog of SidC; anchoring on the LCV by binding to PI(4)P; recruiting Rab10 to the LCV	Bacterial E3 ubiquitin ligases as a paralog of SidC; anchoring on the LCV by binding to PI(4)P; recruiting Rab10 to the LCV	No	No	[158]
49	LubX	*lubX*	N/A	Continuous activating host NF-κB signaling.	Yes, directly	Yes	[159]
50	LamA	*lamA*	N/A	An amylase that can rapidly degrade glycogen to generate cytosolic hyper-glucose, directly triggering an M1-like pro-inflammatory differentiation and directly triggers an M1-like pro-inflammatory differentiation.	Yes, indirectly.	No	[160]
51	Lem27	*lem27*	Dispensable for bacterial intracellular replication	A deubiquitinases regulating LCV Rab10 ubiquitination in concert with SidC and SdcA; dispensable for bacterial intracellular replication in macrophages	No	No	[161]
52	PmrA	*pmrA*	Intracellular proliferation in the ciliate; having a global effect on gene expression with PmrB.	Having a global effect on gene expression with PmrB.	N/A	N/A	[162]
53	PmrB	*pmrB*	Intracellular proliferation; having a global effect on gene expression with PmrA.	Intracellular proliferation; having a global effect on gene expression with PmrA	N/A	N/A	[162]
54	RalF	*ralF*	A guanine nucleotide exchange factor is activated in the presence of the membrane	A guanine nucleotide exchange factor is activated in the presence of the membrane	No	No	[163]
55	LidA	*lidA*	Binding to Rab1 to stabilize the Rab1-guanosine nucleotide complex and activity of the host GTPase Rab1; interfering with the covalent modification of Rab1 by SidD and Lem3	Binding to Rab1 to stabilize the Rab1-guanosine nucleotide complex and activity of the host GTPase Rab1; and interfering with the covalent modification of Rab1 by SidD and Lem3	No	No	[164]
56	LepA and LepB	*lepA* and *lepB*	Playing a role in the non-lytic release of *Legionella* from protozoa	N/A	N/A	N/A	[165]
57	PieE	*pieE*	N/A	Interacting with multiple Rab GTPases; inducing stacked ER membranes; forming complexes with multiple host proteins	N/A	N/A	[147]
58	LppA	*lppA*	A phytate phosphatase (phytase) for efficient replication; promoting intracellular replication in phytate-loaded amoebae	A phytate phosphatase (phytase)	N/A	N/A	[166]
59	Ceg4	*ceg4*	A phosphotyrosine phosphatase; dephosphorylating a broad range of phosphotyrosine-containing peptides	A phosphotyrosine phosphatase; dephosphorylating a broad range of phosphotyrosine-containing peptides; attenuating the activation of MAPK-controlled pathways	Yes, directly.	Yes	[155]
**T2SS effectors**							
1	LapA	* lapA *	An aminopeptidase; playing a role in intracellular replication; generating amino acids for nutrition	An aminopeptidase	N/A	N/A	[73]
2	LapB	* lapB *	Lysine and arginine aminopeptidase	Lysine and arginine aminopeptidase	N/A	N/A	[167]
3	PlaC	*plaC*	A protein with acyltransferase, phospholipase A, and lysophospholipase A activities; playing a dispensable role in intracellular replication	A protein with acyltransferase, phospholipase A, and lysophospholipase A activities; playing a dispensable role in intracellular replication	N/A	N/A	[168]
4	NttA	* nttA *	Contributing to intracellular multiplication in a part of protozoa, such as *Acanthamoeba castellanii*	N/A	N/A	N/A	[169]
5	NttD	* nttD *	Promoting infection of protozoa	N/A	N/A	N/A	[73]
6	NttE	* nttE *	Required for optimal infection of *Acanthamoeba castellanii* and *Hartmannella vermiformis* amoeba	N/A	N/A	N/A	[170]
7	ProA	*proA*	Required for infection of *Hartmannella vermiformis* but not infection of *Acanthamoeba castellanii*	Promoting infection of human lung tissue explants and increasing the alveolar septal thickness; directly degrading immunogenic FlaA monomers; a modulator of flagellin-mediated TLR5 stimulation and the NF-κB pathway	Yes, directly	Yes	[169,171,172]
8	ChiA	*chiA*	A chitinase; not required for intracellular growth;	A chitinase; not required for intracellular growth; directly or indirectly required for optimal survival in the lung tissue	N/A	N/A	[173]
9	SrnA	*srnA*	Required for intracellular infection of specific protozoa, such as *Hartmannella vermiformis*	N/A	N/A	N/A	[169]
**T1SS effectors**							
1	RtxA	*rtxA*	Adherence and entry into host cells, affecting intracellular survival by regulating trafficking	Adherence and entry into host cells, enhancing replication, cytotoxicity, and pore formation	No	No	[90,92]
**Others**							
1	PlaB	*s*	A cell-associated phospholipase A/lysophospholipase A, not essential for intracellular replication	A cell-associated phospholipase A/lysophospholipase A; not essential for intracellular replication; helping bacterial colonization in the lung and enlarging inflammation	N/A	N/A	[174]
2	RsmY	*rsmY*	N/A	Binding to the UTR of ddx58 (RIG-I encoding gene) and cRel, downregulating the expression of Rig-I like miRNA and decreasing the IFN-β response	Yes, directly	Yes	[175]
3	tRNA-Phe	*lppt29*	N/A	Collectively reducing expression of RIG-I, IRAK1 and cRel and downregulating IFN-β	Yes, directly	Yes	[175]

N/A indicates not available.

In conclusion, because of the similarities in bacterial uptake and digestion between protozoa and macrophages, strategies designed to overcome protozoan defenses can be reasonably used to combat the innate immune mechanism in bacteria toward macrophages, and defining proteins that contribute to bacterial entry into eukaryotic cells and intracellular replication in both humans and protozoa as virulence factors may be confusing. The difference between protozoa and human macrophages is whether they have a sophisticated immune system to support them against invasive germs. As a unicellular organism, protozoa only use phagocytosis to resist foreign bacteria, which is a low-level, crude, and ineffective immune strategy that could be easily broken through. On the contrary, the human macrophage, is a member of a highly developed immune system with extensive and potent defense capabilities as well as an advanced regulatory mechanism. Thus, the primary distinction between bacteria-protozoa interaction and bacteria-macrophage interaction is that bacterial infection of macrophages involves more complex interactions between bacteria and the host immune system. Those molecules (e.g., proteins) that aid intracellular survival and replication of *Legionella* by modulating the host immune response can be defined as virulence factors specific to humans. Furthermore, considering human-specific virulence variables in terms of immunological modulation may be more relevant.

## 4. Innate Immune Response to *Legionella*

Being an intracellular pathogen parasite that evolved from a natural protozoan parasite, *Legionella* has developed sophisticated defenses against human macrophage phagocytosis, a crucial part of the first line of innate immune defense. As a result, the immune response via signaling cascades involving infected cell death and inflammatory induction is very important for bacterial clearance and infection control. Like many other bacteria, *Legionella* activates the immune system as soon as it is recognized by first-line immune cells. Once the bacteria invade, several classes of pattern recognition receptors (PRRs) that match pathogen-associated molecular patterns (PAMPs) help macrophages identify foreign bacteria. This recognition triggers the activation of specific intracellular signaling cascades that result in the high production of numerous cytokines and chemokines that are involved in immune responses and inflammation [176]. When *Legionella* infects, several membrane surface and cytosolic PRRs, including Toll-like receptors (TLRs), NOD-like receptors (NLRs), and RIG-I-like receptors (RLRs), recognize bacteria and trigger immune-related signaling pathways [177,178]. The simultaneous and synergistic effects of numerous PRRs recognizing various PAMPs on *Legionella* induce a strong immune response. The innate immune system can restrict *Legionella* replication by promoting the death of infected macrophages and the release of cytokines for the activation of inflammation and adaptive immunity.

The most primary and essential immune initiation is triggered by the recognition of bacterial surface antigen structures, such as cell wall components and the flagellum. A study showed that purified *L. pneumophila* flagellum can induce activation of both innate and adaptive immunity in A/J mice, and the mice group immunized with complete flagellum showed a 100% survival rate when challenged with *L. pneumophila*, demonstrating that the flagellum of *Legionella* as an antigen induces immune memory in mice [179]. In humans, the receptor TLR5 recognizes bacterial flagellin and activates the NF-κB signal with the aid of the adaptor MyD88 [180], and single nucleotide polymorphisms in TLR5 gene sequence are linked to susceptibility to LD [181], thus, TLR5 is a key switch in human immunity to *Legionella*. The fact that flagellum activates innate immunity to *Legionella* in macrophages has also been demonstrated in mouse models. *Legionella* flagellin mutants can overcome innate macrophage defenses, and the Δ*flaA* strains thrive in *naip5* locus-deleted B6 macrophages but not in WT B6 macrophages. However, this process is not dependent on TLR5 but involves the Naip5 protein and caspase-1 to restrict bacterial growth in B6 macrophages [109]. Another common etiology in gram-negative bacteria is LPS. Unlike the majority of gram-negative bacteria, LPS from *L. pneumophila* is primarily recognized by TLR2 but not TLR4 in murine macrophages [182]. This then activates and recruits inflammatory cells, inducing the NF-κB signaling cascade via the MyD88-dependent pathway, and initiating an immune response against the bacterium. [183]. TLR2 has been proven to be crucial for the maturation of several immune cells in mice, including macrophages, granulocytes, and dendritic cells [184,185]. TLR4 is a human receptor for *L. pneumophila* LPS. Using the CRISPR/Cas9 knockout approach, LPS is recognized by different receptors in human macrophages and mouse macrophages [186]. A series of nucleic-acid sensing PRRs, including TLR3, is closely related to optimal cytokine production, including IL-6, IL-10, and certain stimulating molecules when responding to *L. pneumophila* infection. Endosomal TLR3 and TLR4 receptors in human macrophages activate signaling pathways through the adaptor TIR domain-containing adaptor inducing interferon-beta (TRIF), and other cytosolic dsDNA sensors in human macrophages using the cGAS-STING and DNA-PK pathway in response to *L. pneumophila* infection, which is related to cytokine production, such as IL-6 and TNFα [186]. TLR9 is another Toll-like receptor that couples with MyD88; it detects bacterial DNA with an abundance of unmethylated CpG dinucleotides, which is related to the secretion of IL-12 and the production of chemokines as well as type I cytokines but is not essential for cytokine production [187]. TLR4 and TLR5-deficient mice show no impairment in cytokine production or restriction of bacterial replication [182,188], even though these receptors are thought to function in mediating recognition of *Legionella* in alveolar macrophages, and TLR2 and TLR9 are partially required for cytokine production [189,190]. In contrast, MyD88 protein deletion in mice results in increased bacterial replication and decreased cytokine production, indicating that several TLRs work together to cause an effective immune response and may serve a redundant function [191]. The adaptor TRIF of endosomal TLRs functions as a supplement of MyD88 to activate IRF3 and NF-κB. It recruits TRAF6 and activates TAK1 and is responsible for NF-κB activation. TRIF also participates in TLR3-triggered signaling pathways. It activates TAK1 by recruiting the adaptor RIP1 via a homotypic motif, as well as its adaptors TRADD and Pellino-1, and this in turn activates the NF-κB and MAPK pathways [192]. MAPK activation also plays an important role in the host’s immune response to virulent *Legionella*. It was established that *L. pneumophila* infected pulmonary epithelial cells by producing the human antimicrobial peptide β-defensin-2 through a TLR2- and TLR5-mediated pathway, and that MyD88-/T4SS-dependent activation of host MAPK signaling is essential for the best transcription induction of inflammatory cytokines during *L. pneumophila* infection [193,194].

In addition, the cytosolic sensors Nod-like receptors (NLR) help recognize *Legionella*, activate caspase-1 and inflammasome complexes to promote the maturation of the proinflammatory cytokines IL-1β and IL-18, and kill infected macrophages through an inflammatory cell death process known as pyroptosis. For instance, Naip5 (Birc1e) participates in the recognition of cytoplasmic flagellin detection and provides pore-forming activity to cause macrophage death through a separate pathway of TLR-MyD88 signaling [195]. It restricts *L. pneumophila* replication in mouse macrophages by caspase-1 activation, since the level of activated caspase-1 in C57BL/6 macrophages is higher than in A/J macrophages in response to *L. pneumophila* infection, owing to lower expression of Birc1e in the latter [196]. Flagellin participates in both MyD88-mediated signaling and caspase-1-related killing, as a result, distinct signal pathways responding to *Legionella* infection intersect when flagellin is present. Another NLR protein, Ipaf (NLRC4) was demonstrated as important for restricting *Legionella* replication. Macrophages that are deficient in Ipaf can support bacterial growth by increasing 150~1000 folds, and are required for caspase-1 activation in the presence of *Legionella* flagellin, which helps *Legionella*-containing phagosomes mature by acquiring LAMP-1 [197]. Although the adaptor protein Asc, a protein that recruits other proteins with pyrin domains- and caspase recruitment domains via homotypic protein-protein interactions, was proposed to be essential for caspase-1 activation and the secretion of IL-1β and IL-18 through an AIM2, NLRP3, and Ipaf-mediated pathway. It is also suggested that it can take part in an Ipaf/flagellin-independent caspase-1 activation pathway. Because they both release trace amounts of IL-1 and IL-18, like caspase-1-/- macrophages, Asc and Ipaf-mediated pathways may work in tandem to activate caspase-1 [198]. *Legionella* RNA is also an agent that can activate caspase-1 by stimulating Nalp3, one of the components of the inflammasome [199]. In addition, the non-NLR protein AIM2 is a cytoplasmic DNA sensor. It can recognize *Legionella* DNA and recruit the adaptor ASC to activate the inflammasome [200]. However, cytosolic DNA and RNA are difficult to work with because LCV membrane integrity prevents substance leakage in vacuoles, which is maintained by Dot/Icm T4SS substrate SdhA [201]. A non-canonical activation pathway of the inflammasome to cause pyroptosis is dependent on caspase-11 and LPS, which subsequently activate caspase-1 through NLRP3 and ASC, but this pathway requires a functional type IV secretion system [202]. Caspase-11 is not required for the activity of NLRC4-mediated inflammasomes, though it activates caspase-1 in a non-canonical pathway [203]. Besides that, caspase-8 was proven to colocalize with ASC puncta in response to flagellated *L. pneumophila*. Both caspase-1 and caspase-8 were activated by the NAIP5/NLRC4/ASC inflammasome, but caspase-8 was only activated when caspase-1 or gasdermin D were absent [204]. The killing function of NAIP5/NLRC4/ASC inflammasome is partially executed by caspase-7 with a caspase-1/11 independent mechanism [205].

Interferons (IFNs) are major contributors to intrinsic resistance mechanisms in macrophages. Type I and type II IFNs induced by cytosolic sensors such as RIG-I-like receptors, inhibit *Legionella* replication in infected macrophages, by inducing transcription factors to bind to their receptors and enter the nucleus, where they begin the expression of a large number of IFN-stimulated genes (ISGs). A low dose of IFN-α or IFN-β can effectively inhibit *L. pneumophila* multiplication in macrophages, and CD1 mouse-derived macrophages that were originally resistant to *Legionella* became significantly more susceptible after being treated with antibodies to mouse IFN-αβ [206]. Similarly, IFN-γ expression was found to be correlated with anti-*L. pneumophila* activity, and IFN-γ neutralization resulted in increased *L. pneumophila* growth in the macrophages [207]. IFN-γ deficiency increased bacterial recovery in monocyte-derived cells (MC), but had no effects on bacterial reserves in alveolar macrophages and neutrophils [208]. IFN-γ is required for MCs to inhibit *L. pneumophila* replication, but it has no effect on intracellular bacterial multiplication in neutrophils or alveolar macrophages. Alveolar macrophages and neutrophils were demonstrated as primary intracellular reservoirs for *L. pneumophila* in mice with pulmonary infection [209]. Further research indicates that the inability of alveolar macrophages to respond to IFN-γ during *L. pneumophila* infection is due to the downregulation of IFN-γ receptors. The IFN-γ receptor contains IFN-γ receptor subunits 1 and 2 (IFNGR1 and IFNFR2). *L. pneumophila*-infected alveolar macrophages and neutrophils expressed less IFNGR1 than uninfected cells. Preventing IFN-γ receptor down-regulation can reduce *L. pneumophila* intracellular replication in alveolar macrophages *in vivo* [210]. However, the precise mechanism of IFN-γ receptor down-regulation in alveolar macrophages following *L. pneumophila* infection remains unknown. *L. pneumophila* ΔflaA can avoid the attack of the NAIP5 inflammasome and successfully replicate in alveolar macrophages, but IFNs can limit and even completely block the intracellular survival of the bacteria, which is possibly conducted by inducible expression of nitric oxide (NO) synthase to produce NO, and expression of IRG1 via functional IFN signaling to mediate the production of broad-spectrum bactericidal itaconic acid [211,212].

In conclusion, *Legionella* can induce innate immune response through various cell surface and intracellular PRRs, which stimulate many kinds of inflammasomes and multiple redundant signal pathways responsible for the production of inflammatory factors, chemokines, and IFNs. The various kinds of cells and cytokines involved have been reviewed in previous articles [32,213]. Such being the case, how does this kind of bacteria break through multiple barriers of immunity, replicate in various immune cells, and then cause disease in humans? The possible answers relate to its partial evasion of and interference with the host’s immunity.

## 5. Regulation of Host Immune Response by *Legionella*

**Figure 1:**
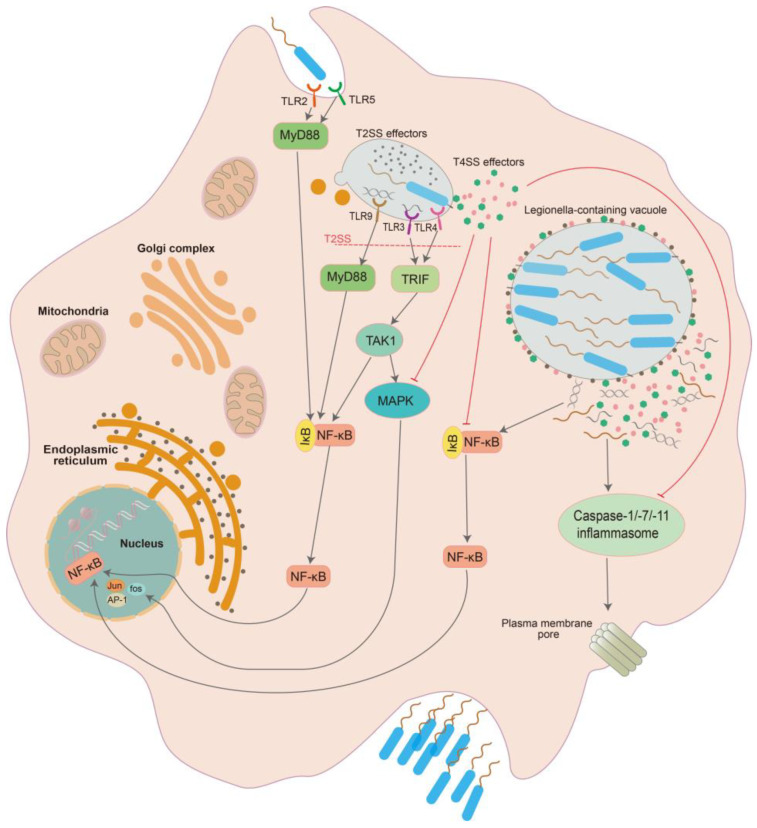
*Legionella* modulates macrophage immunity throughout its life cycle. The schematic shows that *Legionella* suppresses immune activation on macrophages in the early stages of infection and increases it in the late stage to ensure host cell survival and bacterial replication. The black arrow indicates the activation, and the red path indicates the inhibition of the signaling pathway. Once macrophages recognize the LPS and flagellin of *Legionella* bacteria via the pattern recognition receptors TLR2 and TLR5, respectively, immune signaling pathways are instantly activated. T2SS effectors produced by the internalized *Legionella* impair the TLR9-DNA, TLR3-RNA, and TLR4-LPS stimulation of the NF-κB signaling pathway after ingestion, whereas T4SS effectors interfere with the inflammasome activation pathway and then prevent cell pyroptosis. At later phases of infection, decreased LCV membrane integrity leads to increased nucleic acid and flagellin leaks, which collaborate with specific T4SS effectors to continually aggressively stimulate the NF-κB signaling pathway, and considerably drive transcription of inflammatory factors.

The bacteria of *Legionella* can survive in protozoa as a result of escaping the defense mechanisms of single-cell organisms, whereas they have to overcome complex cascades of immune response in a human with a large and intricate immune system if they replicate in alveolar macrophages. That is, some strategies could be used by the bacteria to regulate the host’s immunity and to make the host’s environment conducive to their survival. This is more likely to succeed in populations with compromised immunity, as evidenced by a higher incident rate and more severe symptoms observed in these people [6]. Several bacterial effectors translocated by the T4SS into the cytosol participate in these processes with direct and indirect regulation, which can be roughly classified as positive regulation and negative regulation (Figure 1).

In the early stages of infection, *Legionella* can quickly cause a narrow peak activation of the NF-κB pathway mediated by the LPS/flagellin-PRRs complex as described above. Using a p65-GFP expressing cell to detect NF-κB activation dynamics on a single cell level revealed that the nuclear translocation of p65 increased rapidly in a few minutes of infection [159]. The levels of IκBα in Raw264.7 cells were rapidly decreased to the lowest point at 30 min after infection with *L. pneumophila* when the corresponding activation level of the NF-κB signal reaches the highest point [117]. However, that surge calmed down in a short time as phosphorylatedp65 decayed and IκBα accumulated rapidly. It can be assumed that the unexpected activation suppression is caused by bacterial manipulation. Indeed, *Legionella* employs various strategies to suppress host immunity. It is also known that bacteria can modify intracellular conditions for colonization and replication by partially blocking or isolating immune signaling pathways through membrane proteins or by secreting effectors into the cytoplasm to minimize the immune response. To begin, the protein EnhC has been shown to suppress Nod1-dependent NF-κB activation by inhibiting a transglucosylase of *L. pneumophila* peptidoglycan to regulate the production of PRRs ligands, and Δ*enhC L. pneumophila* strain induced significantly higher NF-κB activation via sltL-dependent method [100]. The expression of EnhC, which is uniquely required for *Legionella* to replicate inside macrophages, is dramatically increased during the post-exponential phase of the organism’s search for a new host [93]. Secondly, the type II secretion system is crucial for reducing the activation of TLR2-triggered NF-κB pathways in various types of human macrophages. This is linked to lower levels of cytokines and chemokines produced by infected cells, such as interleukin 6 (IL-6), tumor necrosis factor-alpha (TNF-α), IL-10, and others [214,215]. An effector protein called ProA is a zinc metalloprotease that is translocated by the type II secretion system and has been demonstrated to suppress the immune response by modifying PPR ligands during the transmissive phase. It can prevent flagellin-TLR5-mediated NF-κB pathway activation by degrading immunogenic FlaA monomers [172,216]. Besides that, several effectors translocated by the T4SS secretion system were proven to participate in host immune signaling inhibition. The transglutaminase MavC, for instance, targets ubiquitin at Gln40 and catalyzes the covalent linkage of ubiquitin to the E2 enzyme UBE2N independent of host ubiquitination mechanisms. Monoubiquitinated UBE2N cannot be used by the canonical ubiquitination machinery, which arrests the degradation of NF-κB signal inhibitor IκBα in the early phase of *L. pneumophila* infection [117]. The effector SdhA, which may join the mitochondria-mediated apoptotic pathway, prevents cell death by preventing DNA leakage from LCV and AIM2 inflammasome-related caspase-1 activation [201]. Another T4SS effector of *L. pneumophila* that contributes to inhibiting host inflammatory signaling is RavD, which was later shown to work as a unique deubiquitinase specialized for linear Ub chains and which inhibits the NF-κB signaling pathway, was originally identified as an inhibitor of endolysosomal maturation [150,151]. RavD inhibits the accumulation of linear Ub chains on LCVs and impairs host NF-κB immune signaling during infection, and a single deletion of the ravD gene can effectively limit *L. pneumophila* multiplication in U937 macrophages [151]. Furthermore, *Legionella* uses post-transcriptional regulation of host gene expression to control host immune signaling pathways, as the bacteria were recently discovered to translocate abundant miRNA-like sRNAs through extracellular vesicles to mimic eukaryotic RNAs and interfere with eukaryotic regulatory mechanisms [175]. The molecules that have been identified are the sRNA RsmY, which, like miRNAs, induces the silencing of *ddx58* (a Rig-I coding gene) and *cRel* (an NF-κB subunit), and the tRNA-Phe, which targets several genes, including several key participants in the recognition of PRRs and their downstream immune signaling, such as *ddx58* and *irak1*, which can collectively downregulate the IFN-β [175]. These results suggest that *Legionella* can utilize eukaryotic-like gene expression regulation to hijack the host immune response. More research is needed to answer the questions of how *Legionella* acquires this mechanism and whether these sRNA also play important roles in protozoa infection.

As stated above, *Legionella* does create compounds that target various immune activation stages, and several of them assume significant roles in the *Legionella* colonization process (e.g., RavD works in escaping endolysosomal maturation pathways). However, this direct regulatory strategy that targets certain molecules in host cells is just one way of regulating host immune activity, and those indirect regulatory strategies may have a more significant effect. While the T4SS effector Lem8 is a protease that is not required for *Legionella* to replicate intracellularly, it inhibits host cell migration by targeting the host regulatory protein 14-3-3ζ Cys-His-Asp motif on the Pleckstrin [217]. The Ran activator LegG1 also modulates phagocyte migration by promoting stabilization and polymerization of microtubules [121,122]. Restricting infected cell migration may impede the spread of chemotactic cytokines and the encounter of healthy immune cells. Inhibition of host protein synthesis is one of the important ways to restrict the potency of the response from the host by blocking the translation of downstream proteins, as well. Fontana et al. demonstrated that at least five Dot/Icm system effectors (Lgt1, Lgt2, Lgt3, SidI, and SidL) were involved in the deduction of protein synthesis in the host because *L. pneumophila* lacking the five genes appeared to compromise the production of several “effector-triggered” genes responsible for the innate immune induction, including Il23a, Gem, and Csf2, but the induction of Ifnb remained intact [218]. The IFN-γ receptor subunit 1 (IFNGR1) is down-regulated in alveolar macrophages and neutrophils after *L. pneumophila* infection, but not in the monocyte-derived cells. Translation inhibition may not only reduce cytokine production from exposed cells but also down-regulate other immune response-related proteins [210]. Alveolar macrophages and neutrophils were previously identified as the primary reservoirs for *L. pneumophila* by tracking infected cells with a tagged T4SS effector [209]. To sum up, *Legionella* uses a variety of tactics, including direct and indirect approaches, to suppress the host cell immune response. This effect appears to be primarily crucial for promptly suppressing immune signals brought on by pathogen-associated molecular patterns (PAMPs) during the first hours of *Legionella* intrusion; thus, early NF-κB activation is strong but transient.

On the contrary, an established infection generally induces a higher level of immune activation in infected human cells. *Legionella* begins to stimulate the NF-κB signaling pathway of host cells again after early infection and keeps it in a relatively high and sustained active state, even up to 30h after infection, during which the activation is dependent on a large number of specific Dot/Icm effectors (e.g., sdbA and lubX) but not the flagellin, TLR5 and MyD88 [159]. A transcriptional profile analysis on U937 cells revealed that when infected by low-dose WT not *dotA*^-^ *L. pneumophila*, a group of proinflammatory cytokines and antiapoptotic genes controlled by the NF-κB were upregulated, with specificity phosphatases 1 (dusp1) being one of the most highly induced genes that help to down-modulate MAPK pathways and prevent cell death. More importantly, dot^+^ *L. pneumophila* infection results in increased cell death and impairs bacterial replication in host cells, indicating that NF-κB translocation regulated by the Dot/Icm effectors is indispensable for *L. pneumophila* survival in host cells [219]. Although a robust activation of caspase-3 was triggered by *L. pneumophila* at early and exponential stages of replication, apoptosis of human macrophages was delayed until the late phases of infection. The anti-apoptotic phenotype has been demonstrated to need sustained activation of NF-κB dependent on Dot/Icm secretion system of *L. pneumophila* in infected macrophages, making infected cells with high caspase-3 activation levels resistant to apoptosis [220]. *L. pneumophila* activates p38 MAPK and NF-κB-dependent expression of a complex group of cytokines when infected by the lung epithelial cells A549 [221]. Activation of both signaling pathways is T4SS-dependent and involved in maintaining the survival of host cells [194,219]. Therefore, vigorously activating the NF-κB signaling pathway by the Dot/Icm secretion effectors is a strategy for *Legionella* to overcome the cell suicide mechanism and maintain its continuous and stable replication by consuming the nutrition of the host cells, and bacterial infection would be prematurely terminated if host cells died via the intrinsic apoptosis pathway.

Several effectors have been demonstrated to activate the NF-κB signal. In total, 13 of 159 *L. pneumophila* T4SS effectors were found to lead to more than a 3-fold increase in NF-κB of HEK293T cells, among which, 11 proteins were mild stimulators represented by VpdA that could cause 14-fold NF-κB activity [119]. The most striking proteins were LegK1 and LnaB, and both of them were dispensable for *L. pneumophila* intracellular growth [119]. LegK1 mimics the host IKK to phosphorylate IκBα and other IκB families of inhibitors such as p100, which is phosphorylated to the mature form of p52 and directly activates the host NF-κB signaling to ~150-fold activity [119,222]. LnaB, a cytoplasm-localized protein that is more highly expressed in *L. pneumophila* than LegK1 displays no homologous sequence with any known protein. It could yield ~120-fold induction of NF-κB in HEK293T cells, which depends on its coiled-coil domain [119]. However, neither LnaB nor LegK1 is indispensable for *L. pneumophila* intracellular growth, even the growth of *L. pneumophila* with Δ*lnaB*Δ*legK1* mutations is slightly more efficient than the wild-type strains, and only Δ*lnaB* can modestly affect NF-κB activity in HEK293T cells. This means that there may be many other redundant effectors dedicated to stimulating NF-κB signaling and knockout of one or two of them does not have an influence on the reduction in bacterial multiplication. Although the exact mechanism is unclear, five effectors involved in translation inhibition were shown to be required for the full effector-triggered response and later sustained activation of NF-κB in an endoplasmic-reticulum (ER) stress-independent way. Certain proteins associated with inflammatory response (e.g., GM-CSF) were produced even when the translation was inhibited [218]. Some specific effector proteins are used to activate the host’s inflammatory response and contribute to *Legionella* replication. However, there is an exception: LamA was identified as an amylase translocated to the cytosol of human macrophages by T4SS from *Legionella*. It can hydrolyze macrophage glycogen and lead to increased cytosolic glucose, which promotes macrophage metabolism entering aerobic glycolysis and starts pro-inflammatory differentiation, and pro-inflammatory cytokine secretion, which partially restricts *Legionella* proliferation in macrophages [160]. In viewing the counter-evolutionary function of LamA in limiting *Legionella* replication within macrophages and its presence in all analyzed *L. pneumophila* strains, the amylase was speculated to have been accidentally acquired from *Acanthamoebae* by inter-domain horizontal gene transfer, in which LamA subverts amoeba encystation [160]. In conclusion, although suppression of cellular immune responses in the early infection stage is important for bacterial colonization, activation of MAPK and NF-kB for maintaining cell viability is required for continued bacterial replication. These responsive signaling pathways produce massive amounts of proinflammatory cytokines that promote inflammation and are essential for initiating adaptive immunity, whereas intense inflammatory reactions are the direct cause of inflammatory diseases such as pneumonia by causing tissue damage and lung dysfunction.

## 6. *Legionella* Virulence Is Related to the Early Immunosuppressive and Later Inflammatory Levels in the Host

*Legionella*, a human pathogen that evolved from its environment, regulates host cell immune responses to adapt to the intracellular and internal environments of new hosts. The difference in the ability of *Legionella* to regulate the immune response of host cells may be a reason for the heterogeneity of *Legionella* virulence. It is described above that *Legionella* can inhibit the immune response of host cells after infection. Other researchers have found that early activation of innate immunity with IFN-γ from pre-infection to 2 h after infection can significantly inhibit *Legionella* replication in host cells [206,223,224,225]. To state it another way, the bacteria’s negative regulation of host immunity has a significant impact on infection success. Due to lower flagellin expression compared to the planktonic form of *L. pneumophila*, biofilm-derived *L. pneumophila* can evade caspase-1 and caspase-7-mediated innate immune response, resulting in reduced bacteria-containing vesicles and lysosome fusion and low host cell mortality, as well as more effective bacterial replication within murine macrophages [226]. It has been previously demonstrated that NLRC4/NAIP5-mediated caspase-1 and caspase-7 activation regulates host cell death by both pyroptosis and autophagy means and facilitates bacterial degradation by stimulating the fusion of *Legionella*-containing phagosomes and lysosomes within 2 h after infection [197,227,228]. Therefore, bacteria that express less flagellin have more potential to overcome the host cells’ innate immune system defense. Comparing the expression difference in the virulence gene of *L. pneumophila* infected with protozoa and macrophages, it was discovered that *flaA*, which is involved in inducing host cell death, was downregulated during the infection with THP-1, whereas the *sdhA* gene, which maintains LCV membrane integrity, was substantially upregulated. In contrast, a higher expression of *flaA* and *vipD* genes that induce apoptosis, and a lower expression of *sdhA* and *sidF* that suppress apoptosis gene expression were observed in the late stage of *L. pneumophila* replication within protozoa [229]. *L. longbeachae* and *L. pneumophila* are the most prevalent and lethal agents in the genus *Legionella*. Compared with *L. pneumophila*, *L. longbeachae* fails to activate the Naip5/Nlrc4 inflammasome in mice due to the lack of flagellar biosynthesis genes [52] *L. longbeachae* has been shown to induce less systematical production of inflammatory cytokines (IL-12, TNF-α, and IL-8) in mouse and human macrophages than *flaA*^-^ *L. pneumophila* and cannot cause sepsis in mice, both of which are important for the resistance of *L. longbeachae* infection by reducing bacterial load. Therefore, *L. longbeachae* can robustly multiply in the lung and induce severe pulmonary injury, leading to lethal lung dysfunction [230]. Even flagellated *L. longbeachae* was previously demonstrated to fail to trigger caspase-1 activation and pore-forming activity in mouse or human macrophages, only modest caspase-3 and cell apoptosis were observed at late stages of infection [231]. Furthermore, the results of the mouse infection model with *L. longbeachae* and *L. pneumophila flaA-* suggested that mice infected with *L. longbeachae* but not with *L. pneumophila* had more severe lung injury, weight loss, and higher mortality, which could be attributed to reduced induction of cytokines and host innate immunity due to the absence of the flagellum and other immune stimuli [230]. Therefore, weakening the immune response of host cells in the early stages of infection is beneficial and crucial for *Legionella* colonization and replication.

*Legionella* virulence is displayed by the strength of the immune response during the later stages of infection, and the virulent strain tends to elicit a strong inflammatory response. After being infected with virulent and avirulent *L. pneumophila* strains, the murine macrophages of A/J mice did produce differential levels of proinflammatory cytokines at 24 h and 48 h after infection. High levels of TNFα, IL-1α, IL-1β, and IL-6 were presented at both time points in the culture supernatant of macrophages infected with the virulent strain, while these cytokines were undetectable in the group infected with avirulent strains [232]. *Legionella* regulates not only the production of various proinflammatory cytokines but also certain cytokine receptors to enhance the susceptibility of host cells to infection since IL-1 and TNF receptors in A/J mouse macrophages were likewise up-regulated by virulent but not avirulent *L. pneumophila* [233]. The virulent *L. pneumophila* M124 strain was shown to induce expression of genes related to inflammation at 5h and markedly increase at 24h after infection, including monocyte chemotactic protein 3 (MCP-3) and macrophage inflammatory protein 1α (MIP-1α). Both are involved in monocyte chemotaxis, but the avirulent bacteria did not induce MCP-3 [234]. *L. pneumophila* induces NF-κB activation with the biphasic stimulation method. The phase I activation of immune response is mediated by PRRs, which would be inhibited within 3 or 6 h after infection, and the subsequent sustained phase II activation is mainly contributed by effector proteins [159]. Therefore, the production of proinflammatory cytokines described above is related to effector proteins, because effectors secreted by the avirulent *L. pneumophila* strain fail to activate and sustain host immune responses. Consistently, avirulent strains can survive but cannot multiply in macrophages, which are different from those virulent strains, but the bacteria’s quantity barely increased by 24h after infection [232,234]. It is unclear whether there is a link or causal relationship between bacterial replication ability and immune stimulation. However, according to the study by Wang et al., the ability of bacteria to activate and maintain host inflammatory responses is positively correlated with their virulence to the host. This study shows that the *L. pneumophila* strain with a stronger stimulation ability of NF-κB signaling can cause higher concentrations of some cytokines (such as IL-2, IL-5, IL-6, etc.) and more severe lymphocyte infiltration in the lung tissue, as well as more weight loss and mortalities in mice [31]. In a comparative analysis of virulence for two *L. feeleii* strains referred to as LfPF and LfLD which cause Pontiac fever and Legionnaires’ disease, respectively, the results showed that LfLD had superiorities in mobility and high-temperature resistance, bacterial internalization, and growth in host cells, cytotoxicity to host cells and induction ability of IL-6 and IL-8 cytokines, and was even superior to the positive control *L. pneumophila* JR32 [26]. Some sequence types (ST) such as ST1, ST23, and ST47 are more likely to cause infection in humans and are frequently found in clinical samples. Based on 108 *L. pneumophila* sg1 isolates from the clinic, an analysis of three STs revealed that U937 macrophages infected by ST1 strains and ST47 strains produced significantly higher and lower TNF-α secretion, which were mediated by the NF-κB signaling pathway and were associated with host cell death as a result of higher lactate dehydrogenase (LDH) and caspase-3 activities induced by ST1 rather than ST47 isolates [235]. The differences in TNF-α secretion between ST1 and ST47 strains corresponded to their clinical occurrence, as ST1 strains were more frequently isolated from immunosuppressed patients suffering from cancer/malignancy than ST47 strains [235]. Thus, those bacteria that can strongly and sustainably stimulate an immune response and an inflammatory reaction are more virulent and dangerous to humans.

## 7. Conclusions

The pathogenicity of *Legionella* is an unexpected result of evolution in which the bacteria continuously adapt to various environmental hosts, and the same process and mechanism are utilized to infect various protozoa. Proteins involved in the infection process of protozoa cells, including bacterial invasion, host trafficking regulation, LCV establishment, and intracellular replication of bacteria, were previously defined as “general virulence factors”. However, a large number of them assume important responsibilities in infecting their environmental host protozoa and are indispensable for *Legionella* survival, and it would be inaccurate to define those proteins as virulence factors of *Legionella* from the human perspective. On the contrary, distinguishing human- or protozoa -specific virulence factors based on the essential difference between an environmental host and an accidental host may be a more precise difinition. Whether there is a huge immune regulatory system as a support to defense against bacterial infection is the main difference between an environmental host and an accidental host, such as humans, and the former is one of the members of the protozoa immune system and plays crucial roles in the processes of innate and acquired immunity. *Legionella* has to contend with the host immune regulatory system when infecting macrophages; indeed, hindering the normal immune response of the host is necessary for successful infection. The bacteria of *Legionella* inhibit the host immune response to evade immune attack at the early stages of infection and sustainably activate inflammation and fully utilize nutrients in host cells at the late stages of infection (Figure 1). In terms of pathogenesis, early immune suppression provides an opportunity for intracellular colonization of the bacteria, while the strong inflammatory response at the later stage is the actual cause of the human diseases. Thus, in addition to *Legionella*’s intracellular replication and infection capacity, interfering with host immune regulation is a key factor in a successful infection. Accordingly, the capacity of *Legionella* to regulate host immunity may also be logically presented as its capacity for intracellular replication. Proteins or factors that directly participate in those regulations can be defined as virulence factors specific to humans. More virulence factors involved in regulating the host immune response may be revealed by screening proteins with varied expression in the early and late phases of infection. Comparing the ability of different *Legionella* strains to control human immunity, which could be caused by immune regulation-related protein composition and polymorphism, could also help to explain the variation in virulence among them.

## Data Availability

No data set is linked to this article.
